# Life-Space Constriction in Aging Adults

**DOI:** 10.1093/geroni/igab046.2359

**Published:** 2021-12-17

**Authors:** Tamatha Arms

**Affiliations:** UNC Wilmington, Wilmington, North Carolina, United States

## Abstract

Community-dwelling aging adults desire to maintain independence and prevent or delay a sequela of declining function and ultimate frailty. Early indicators of potential declines in function and frailty, such as life-space constriction (LSC), are important to identifying early. The purpose of this study was to examine factors associated with LSC and the influence of these factors and LSC on function and frailty. A cross-sectional study using a convenience sample of community dwelling persons 55 and older living in the South was conducted. Results indicated most participants (N = 72) were female (69%; n = 50) and half were White (53.5%; n = 38). LSC explained 34% variance in function (F = 3.805 (8, 59); p = .001) when environmental supports (social network), challenges (driving time it took the participant to get to the nearest full-service grocery store) and individual factors were controlled for. There was a significant difference between Black and White participants with environmental challenges (p = .001) and function (p = .001). Individual factors included challenges (age-related physiological changes, disease burden, and mental health limitations) and buoy (assistive devices), these explained 22% variance in self-reported frailty (F= 3.099 (6, 65); p = .01). Number of assistive devices was the only significant predictor of self-reported frailty.

